# High-dose vitamin D versus placebo to prevent complications in COVID-19 patients: A structured summary of a study protocol for a randomised controlled trial (CARED-TRIAL)

**DOI:** 10.1186/s13063-021-05073-3

**Published:** 2021-02-01

**Authors:** Javier Mariani, Carlos Tajer, Laura Antonietti, Felipe Inserra, León Ferder, Walter Manucha

**Affiliations:** 1 Hospital de Alta Complejidad en Red El Cruce - Néstor Kirchner, Florencio Varela, Buenos Aires, Argentina; 2 Universidad Nacional Arturo Jauretche, Florencio Varela, Buenos Aires, Argentina; 3 Maimónides University, Ciudad Autónoma de Buenos Aires, Argentina; 4 Consejo Nacional de Investigaciones Científicas y Técnicas, Universidad Nacional de Cuyo, Instituto de Medicina y Biología Experimental de Cuyo (IMBECU), Mendoza, Argentina

**Keywords:** COVID-19, Randomised controlled trial, protocol, and vitamin D, SARS-CoV-2, mechanical ventilation, pneumonia

## Abstract

**Objectives:**

To evaluate whether a single high dose of oral cholecalciferol improves the respiratory outcomes as compared with placebo among adults COVID-19 patients at moderate risk of clinical complications.

**Trial design:**

The CARED trial is an investigator-initiated, multicentre, randomized, parallel, two-arm, sequential, double-blind and placebo-controlled clinical trial. It was planned as a pragmatic trial since the inclusion criteria are broad and the study procedures are as simple as possible, in order to be implemented in the routine clinical practice in general wards in the pandemic setting and a middle-income country context. The sequential design involves two stages. The first stage will assess the effects of vitamin D supplementation on blood oxygenation (physiological effects). The second stage will assess the effects on clinical outcomes.

**Participants:**

Participants of either gender admitted to general adult wards in 21 hospital sites located in four provinces of Argentina are invited to participate in the study if they meet the following inclusion criteria and none of the exclusion criteria:

*Inclusion criteria*

- SARS-CoV-2 confirmed infection by RT-PCR;
- Hospital admission at least 24 hours before;
- Expected hospitalization in the same site ≥24 hours;
- Oxygen saturation ≥90% (measured by pulse oximetry) breathing ambient air;
- Age ≥45 years or at least one of the following conditions:
  - ○ Hypertension;
  - ○ Diabetes;
  - ○ At least moderate COPD or asthma;
  - ○ Cardiovascular disease (history of myocardial infarction, percutaneous transluminal coronary angioplasty, coronary artery bypass grafting or valve replacement surgery);
  - ○ Body mass index ≥30;
- Willingness to sign informed consent (online supplementary material 1 and 2).

*Exclusion criteria:*

- Age <18 years;
- Women in childbearing age;
- >= 72 hs since current admission;
- Requirement for a high dose of oxygen (>5 litres/minute) or mechanical ventilation (non-invasive or invasive);
- History of chronic kidney disease requiring haemodialysis or chronic liver failure;
- Inability for oral intake.
- Chronic supplementation with pharmacological vitamin D;
- Current treatment with anticonvulsants;
- History of:
  - ○ Sarcoidosis;
  - ○ Malabsorption syndrome;
  - ○ Known hypercalcemia or serum calcium >10.5 mg/dL;
- Life expectancy <6 months;
- Known allergy to study medication;
- Any condition at discretion of investigator impeding to understand the study and give informed consent.

**Intervention and comparator:**

The intervention consists in a single oral dose of 500.000 IU of commercially available cholecalciferol soft gel capsules (5 capsules of 100.000 IU) or matching placebo

**Main outcomes:**

The primary outcome for the first stage is the change in the respiratory Sepsis-related Organ Failure Assessment (SOFAr) score between pre-treatment value and the worst value recorded during the first 7 seven days of hospitalization, the death or discharge, whichever occurs first. The SOFAr score measured as the ratio between the pulse oximetry saturation (SpO_2_) and FiO_2_ (27, 28) is used instead of the arterial partial pressure of oxygen (PaO_2_). SOFAr score is a 4-points scale, with higher values indicating deeper respiratory derangement as follows: 1 PaO_2_ <400; 2 PaO_2_ <300; 3 PaO_2_ <200; 4 PaO_2_ <100.

The primary outcome for the second stage is the combined occurrence of requirement ≥40% of FiO_2_, invasive or non-invasive ventilation, up to 30 days or hospital discharge.

**Randomisation:**

A computer-generated random sequence and the treatment assignment is performed through the web-based randomization module available in the electronic data capture system (Castor®). A randomization ratio 1:1, stratified and with permuted blocks was used. Stratification variables were diabetes (yes/no), age (≤60/>60 years) and the site.

**Blinding (masking):**

Double-blind was achieved by using placebo soft gel capsules with the same organoleptic properties as the active medication. Central management of the medication is carried out by a pharmacist in charge of packaging the study drug in unblinded fashion, who have no contact with on-site investigators. Medication is packaged in opaque white bottles, each containing five soft gel capsules of the active drug or matching placebo, corresponding to complete individual treatment. Treatment codes are kept under the pharmacist responsibility, and all researchers are unaware of them.

**Numbers to be randomised (sample size):**

The first stage is planned to include 200 patients (100 per group), the second stage is planned to include 1064 additional patients. The total sample size is 1264 patients.

**Trial Status:**

Currently the protocol version is the number 1.4 (from October 13^th^, 2020). The recruitment is ongoing since August 11th, 2020, and the first subject was enrolled on August 14th. Since then, 21 sites located in four provinces of Argentina were initiated, and 167 patients were recruited by January 11^th^, 2021. We anticipate to finish the recruitment for the first stage in mid-February, 2021, and in August, 2021 for the second stage.

**Trial registration:**

The study protocol is registered in ClinicalTrials.gov (identifier number NCT04411446) on June 2, 2020.

**Full protocol:**

The full protocol is attached as an additional file, accessible from the Trials website (Additional file 1). In the interest in expediting dissemination of this material, the familiar formatting has been eliminated; this Letter serves as a summary of the key elements of the full protocol.

The study protocol has been reported in accordance with the Standard Protocol Items: Recommendations for Clinical Interventional Trials (SPIRIT) guidelines (Additional file 2).

**Supplementary Information:**

The online version contains supplementary material available at 10.1186/s13063-021-05073-3.

## Supplementary Information

**Additional file 1.** Protocol.

**Additional file 2.** SPIRIT 2013 Checklist: Recommended items to address in a clinical trial protocol and related documents*.

## Data Availability

Authors will have access to the final dataset. Data will be available from the corresponding author upon reasonable request.

