# Supplementary material for: High-dose vitamin D versus placebo to prevent complications in COVID-19 patients: A structured summary of a study protocol for a randomised controlled trial (CARED-TRIAL)
Source: Trials. 2021 Feb 1;22:111. doi: 10.1186/s13063-021-05073-3 (PMC7848249; doi:10.1186/s13063-021-05073-3)
Supplement: Supplementary file 1 — Additional file 1. Protocol. [file 13063_2021_5073_MOESM1_ESM.pdf]

Ensayo controlado con vitamina D en altas dosis versus placebo para prevenir las complicaciones evolutivas de pacientes infectados por COVID-19

Nombre corto: **C**olec**A**lcife**R**ol para m**E**jorar la evolución de pacientes con COVI**D**-19 (**CARED**)

Investigadores: Walter Manucha[1], Leon Ferder[2], Felipe Inserra[2], Javier Mariani[3,4], Laura Antonietti[3,4] ,Carlos Tajer[3,4] .

Filiación:

[1] Instituto de Medicina y Biología Experimental de Cuyo (IMBECU), CCT-Mendoza, CONICET, Facultad de Ciencias Médicas, Universidad Nacional de Cuyo, Av. Ruíz Leal, s/n Parque Gral. San Martín (5500), Mendoza, Argentina.

[2] Universidad Maimónides, Hidalgo 775 (C1405), Ciudad Autónoma de Buenos Aires, Argentina.

[3] Hospital de Alta Complejidad El Cruce Néstor Kirchner, Av. Calchaquí 5401 (C1888), Florencio Varela, Buenos Aires, Argentina.

[4] Universidad Nacional Arturo Jauretche, Av. Calchaquí 6200 (C1888), Florencio Varela, Buenos Aires, Argentina.

Confidencial

1. Introducción

Un nuevo coronavirus (SARS-CoV-2) ha generado una pandemia con una gran cantidad individuos infectados y muertos a nivel global por las complicaciones respiratorias del virus (1).

El SARS-CoV-2 provoca la enfermedad COVID-19 ingresando a las células del epitelio alveolar por su afinidad por los receptores de la enzima convertidora de angiotensina ACE-2 (2). La reducción de la actividad de la enzima ACE-2 inducida es un mecanismo patogénico de los coronavirus a nivel pulmonar, por exacerbación de la actividad local de la angiotensina II que induce una activa inflamación (3). En los primeros reportes evolutivos de pacientes infectados por COVID-19, el mayor riesgo de complicaciones y mortalidad se asoció a edad avanzada, hipertensión, diabetes, enfermedades pulmonares y cardíacas. Estas condiciones clínicas tienen exaltación del sistema renina-angiotensina (RAS) (4-8) con un bajo nivel de vitamina D (9). Coincidentemente, las complicaciones pulmonares y las muertes en infecciones virales tienen una relación inversa con los niveles séricos de vitamina D (10-14). Múltiples estudios muestran que la vitamina D tendría un efecto antagónico sobre el RAS a nivel tisular, reduciendo la respuesta inflamatoria (9, 15-23). En este contexto el tratamiento para aumentar los niveles de vitamina D podría ser beneficioso.

Nuestro supuesto se fundamenta en evidencia propia y de otros autores, que aportan información epidemiológica y clínica de la modulación de la vía inflamatoria en infecciones virales graves por la vitamina D, con la particularidad de la interacción del COVID-19 sobre la enzima ACE2 y la modulación que ejerce la vitamina D sobre la misma. Por lo tanto, suplementar vitamina D podría brindar protección contra COVID-19, tanto en la prevención como en el desarrollo y/o evolución de la enfermedad (Figura 1 y 2). Dados estos antecedentes y la magnitud de la pandemia se torna imprescindible explorar el rol de la vitamina D para prevenir y disminuir las complicaciones de la infección COVID-19 (24, 25).

Se ha demostrado que la administración de una única dosis elevada de vitamina D aumenta los niveles sanguíneos de 25 (OH) vitamina D a niveles suficientes y estos niveles se mantienen elevados durante más de un mes; estos efectos terapéuticos se

Confidencial

logran con una mínima incidencia de eventos adversos leves (26). El uso de una dosis única de 500.000 UI de colecalciferol se justifica por la pertinencia de alcanzar niveles séricos de vitamina D adecuados en un breve período de tiempo desde el inicio de la infección por SARS-CoV-2. Además, considerando la dinámica de la COVID-19 y sus potenciales complicaciones respiratorias, una dosis única permitiría cubrir todo el período de apación de mayor riesgo con un excelente perfil de seguridad. Aunque existe cierto debate sobre posibles efectos tóxicos, estos se han reportado principalmente con la administración crónica de la vitamina D, sin que haya reportes suficientes de toxicidad importante con el uso de una dosis única alta, mientras que existe una vasta evidencia científica de efectividad, carente de efectos tóxicos hasta el momento.

El objetivo del estudio es evaluar si la administración de vitamina D en altas dosis es una intervención efectiva para disminuir la tasa de complicaciones respiratorias y sistémicas en pacientes con infección COVID-19 confirmada.

Figura 1: Vías de señalización en los potenciales efectos terapéuticos de la vitamina D en la enfermedad COVID-19.

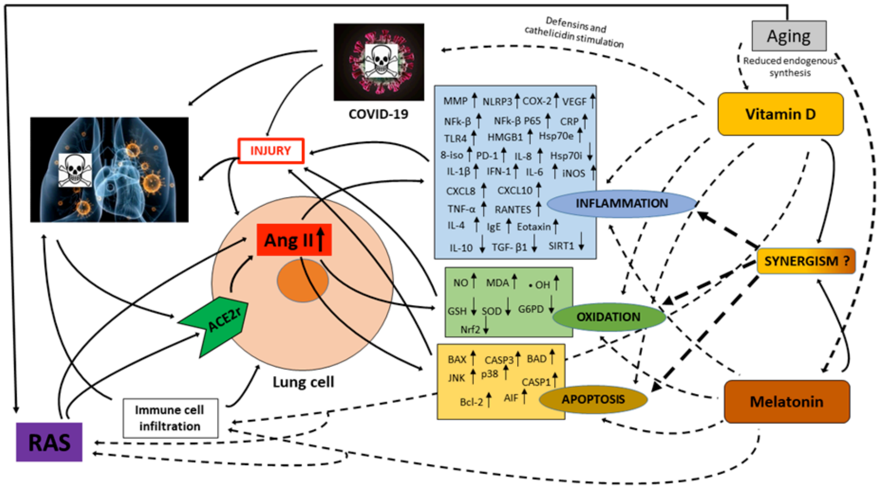

Resumen gráfico de las principales vías de señalización comunes entre la melatonina y la vitamina D como un nuevo tratamiento sinérgico potencial en la infección pulmonar por COVID-19. A los efectos del presente proyecto, se desean destacar aquellas vías que por evidencia científica involucran regulación/modulación entre el sistema RAS y la vitamina D, dos protagonistas racionales en la infección y evolución del COVID-19. Las líneas continuas indican estimulación/inducción, mientras que las líneas discontinuas indican inhibición/bloqueo.

(27) Lungs as Target of COVID-19 Infection: Protective Common Molecular Mechanisms of Vitamin D and Melatonin as a New Potential Synergistic Treatment. Gimenez M, Inserra F, Tajer C, Mariani J, Ferder L, Manucha W. Aceptado para publicación Life Sciences, 2020.

Figura 2: Interacción entre el sistema renina-angiotensina y la vitamina D (potenciales implicancias terapéuticas).

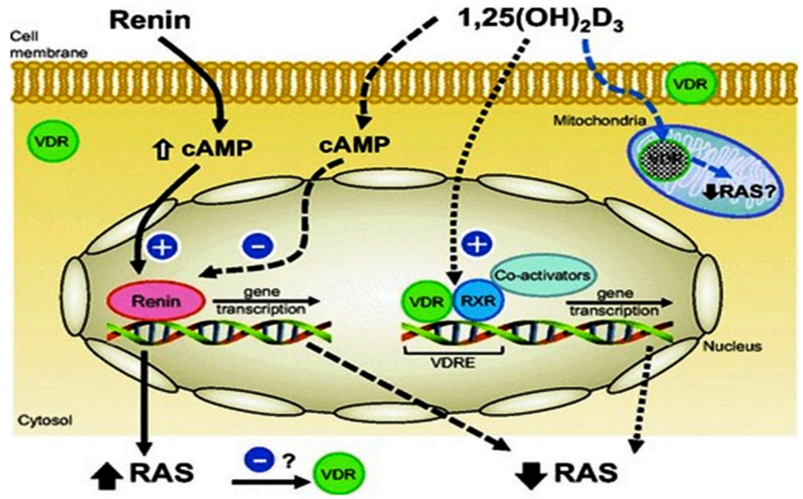

Interacciones celulares de angiotensina y receptores de Vitamina D. RXR, receptor retinoide X; RAS, sistema renina-angiotensina; VDRE, elemento de respuesta Vitamina D; 1,25 $(OH)_2D3$, 1,25-dihidroxivitamina D3. (Ferder, Inserra, Manucha. Am J Physiol Cell Physiol. 2013 Jun 1; 304(11): C1027–C1039).

2. Material y métodos

a. Diseño

Se diseñó un ensayo clínico de Fase IV, aleatorizado, multicéntrico, doble ciego, de grupos paralelos, secuencial, controlado con placebo, para evaluar los efectos de una alta dosis de vitamina D sobre la evolución de pacientes con diagnóstico confirmado de infección COVID-19.

b. Participantes

Se considerarán para el estudio los individuos que cumplan los siguientes criterios de inclusión y ninguno de los de exclusión:

- **Criterios de inclusión**
  - Confirmación por laboratorio del virus SARS-CoV-2 a través de PCR o la metodología estándar que utilice el centro;
  - Estar internado/a en un centro asistencial;

Confidencial

- Internación esperada en el mismo centro de al menos 24 hs;
- Saturación arterial de oxígeno mayor a 90% respirando aire ambiente;
- Edad ≥45 años y/o al menos un criterio asociado a mayor riesgo de complicaciones evolutivas por COVID-19: Hipertensión arterial, Historia de diabetes I o II, Antecedentes de asma/EPOC, Antecedentes cardiovasculares (historia de infarto de miocardio, angioplastia coronaria, cirugía de revascularización o de reemplazo valvular), Obesidad (índice de masa corporal ≥30);
- Disposición a firmar el consentimiento informado del estudio.

▪ **Criterios de exclusión**

- Edad menor a 18 años
- Haber permanecido internado/a ≥72 horas en cualquier centro asistencial
- Mujeres en edad fértil (si al momento de la evaluación ha transcurrido menos de un año desde la fecha de última menstruación);
- Requerimiento oxigenoterapia de alto flujo, asistencia respiratoria no invasiva (VNI) o invasiva (asistencia respiratoria mecánica);
- Insuficiencia renal crónica en diálisis o insuficiencia hepática conocida;
- Intolerancia oral;
- Tratamiento farmacológico crónico con vitamina D;
- Historia de:
    - Tratamiento con cualquier medicación anticonvulsivante;
    - Sarcoidosis;
    - Síndromes de mala absorción;
    - Hipercalcemia conocida o Calcemia superior a 10,5 mg/dL.
- Enfermedad sistémica considerada terminal (expectativa de vida menor de seis meses);
- Alergia conocida a la medicación del estudio;
- Cualquier condición que impida brindar el consentimiento informado.

c. Intervenciones

Confidencial

Se administrará una dosis única de 500.000 UI de Vitamina D_3 (colecalciferol) o placebo. La medicación del estudio consistirá en 5 cápsulas blandas conteniendo 100.000 UI de Vitamina D o placebo cada una. Con esta dosis de Vitamina D_3 se han observado niveles suficientes de 25 (OH) vitamina D en plasma ya a las 24 hs y su persistencia dentro del rango >29 ng/ml más allá del mes de su administración (26).

d. Puntos finales

El protocolo tiene un diseño secuencial, en el cual se evaluará un primer objetivo relacionado con la evolución de la oxigenación. En caso de obtener un resultado favorable en el análisis interino, se ampliará el reclutamiento de pacientes y se evaluarán objetivos adicionales.

En la segunda etapa del estudio, el primer objetivo secundario de la primera etapa pasará a ser un punto final combinado principal de eficacia.

- **Primera etapa**

  - **Punto final Primario**

Cambio en el puntaje SOFA respiratorio entre el ingreso y el peor SOFA respiratorio registrado (se utilizará la saturación arterial de O_2 medida por oxímetro de pulso para estimarla) (28).

  - **Puntos finales Secundarios**

Los objetivos secundarios del trabajo serán analizados hasta el egreso de la internación.

1) Cambio en la saturación de O_2 entre el ingreso y la peor saturación de O_2 registrada.
2) Desaturación: saturación de O_2 menor o igual al 90% respirando aire ambiente en cualquier momento de la internación, medida por oxímetro de pulso.
3) Punto final combinado: requerimiento de asistencia con oxigenoterapia de alto flujo (>40% de Fi O_2), indicación de ventilación no invasiva o invasiva.
4) Cambio en el puntaje quick SOFA desde el ingreso al peor puntaje registrado durante la internación.
5) Incidencia combinada de accidente cerebrovascular, insuficiencia renal aguda, infarto de miocardio y/o tromboembolismo pulmonar.
6) Derivación a cuidados intensivos por necesidades clínicas.

Confidencial

7) Ingreso a ventilación mecánica invasiva.
8) Días de internación total.
9) Días de internación en terapia intensiva.
10) Mortalidad.

- **Segunda Etapa**

Si se obtienen resultados favorables al finalizar la primera etapa y por decisión del Comité ejecutivo del ensayo, se pasará a la segunda etapa del protocolo.

- **Punto final Primario**

Evento combinado: requerimiento de asistencia con oxigenoterapia de alto flujo (>40% de Fi O_2) , indicación de ventilación no invasiva o invasiva.

- **Puntos Finales Secundarios**

1) Derivación a la Unidad de Cuidados Intensivos por necesidades clínicas.
2) Ingreso a ventilación mecánica invasiva.
3) Días de internación total.
4) Días de internación en Cuidados Intensivos.
5) Mortalidad.

e. Subestudio fisiopatológico:

A los fines de un análisis fisiopatológico, en algunos centros seleccionados de la Ciudad de Buenos Aires y la Provincia de Buenos Aires, en un subgrupo de pacientes enrolados en el estudio se tomará una muestra sanguínea al ingreso y otra muestra sanguínea entre los días +3 a +7, o el día del egreso hospitalario (lo que ocurra primero). Una alicuota de las muestras será destinada a valorar parámetros de actividad inflamatoria, del sistema renina angiotensina y los niveles alcanzados de OH vitamina D en plasma. (Ver ANEXO SUBESTUDIO FISIOPATOLÓGICO CARED)

El remanente de las muestras se almacenará para eventuales mediciones que en el futuro resultaran de interés científico. Todas las muestras serán almacenadas de manera codificada a los fines de garantizar la confidencialidad de los datos de los pacientes. Previo a la toma de muestras, a cada paciente que haya aceptado participar se le invitará

Confidencial

a participar en el subestudio fisiopatológico y se le solicitará que brinde un consentimiento específico para dicho subestudio. (Ver Anexo CONSENTIMIENTO INFORMADO DEL SUBESTUDIO FISIOPATOLÓGICO).

f. Recolección de la información

Toda la información será ingresada a un formulario de reporte de casos electrónico (eCRF, por sus siglas en inglés) con datos encriptados y anonimizados. La base de datos será construida *ad hoc* para el ensayo clínico y cumplirá con la legislación vigente acerca de protección de datos personales (Ley 25.326).

Toda la información con respecto a los sujetos de estudio será guardada en archivos de computadora, protegidos con contraseña o en armarios bajo llave a los que tendrán acceso solamente del personal autorizado del estudio. Las muestras biológicas, tablas y archivos serán identificadas por el número único de identificación. Los datos de los cuestionarios y fichas clínicas serán ingresados en la base de datos. Semanalmente se realizará un back up de la base de datos y se guardarán en duplicados en un servidor al que solo tiene acceso el Comité Ejecutivo del estudio.

Se guardarán las muestras biológicas para investigaciones futuras durante un período máximo de 15 años, y se podrán analizar solo con fines científicos. El consentimiento informado específico del subestudio incorpora este ítem, explicitando el derecho de los participantes de comunicarse en cualquier momento con el investigador principal para que las mismas sean destruidas y dejen de ser utilizadas para eventuales análisis, si así lo desearan.

Confidencial

g. Seguimiento

El seguimiento de los pacientes estará limitado a la internación. Se registrarán los puntos finales fisiopatológicos (saturación de O_2) hasta el séptimo día, la muerte o el alta hospitaria, lo que ocurra primero. Los puntos finales clínicos se registrarán hasta la muerte, el alta o el mes, lo que ocurra primero.

h. Procedimientos del estudio

El/La investigador/a principal de cada centro u otros investigadores designados por éste/a evaluarán los criterios de inclusión/exclusión de potenciales participantes en el estudio.

En caso de cumplir los criterios de inclusión y ninguno de los de exclusión se le invitará a participar del ensayo. Tras la obtención del consentimiento informado, el/la investigador/a del centro recolectará y registrará en el CRF la información basal del paciente al momento de su inclusión: Tº axilar, presión arterial (PA), frecuencia cardíaca (FC) y respiratoria (FR), saturación de O2 (Sat. O2), fracción inspirada de O2 (FiO2) y si recibe O2 suplementario. Se registrarán además datos relativos a síntomas de COVID-19 y antecedentes clínicos relevantes, incluyendo tratamiento habitual del/de la paciente, el nivel de calcemia y otros datos de análisis clínicos de laboratorio - si se hubiera realizado análisis de rutina (Ver Anexo Formulario de Reporte Clínico).

Para la medición de la saturación de O_2 basal, el/la paciente deberá estar respirando aire ambiente durante al menos 5 minutos hasta alcanzar una oximetría de pulso en estado estable (29).

Luego se realizará la aleatorización electrónica del paciente, a través del eCRF. El/La investigador/a registrará -tanto en el eCRF como en la historia clínica- el grupo de tratamiento y el número de envase asignado, administrará la medicación del estudio y presenciará la ingesta de la misma.

Diariamente los integrantes del equipo de investigación registrarán: Tº axilar, signos vitales (PA, FC, FR), la mínima saturación de O_2 por oximetría de pulso y la fracción inspirada de O_2 en el momento de la medición (FiO_2). Dichos datos se registrarán durante los primeros siete días, hasta la muerte o el alta, lo que ocurra primero. La saturación respirando aire ambiente, tanto al ingreso como en cada evaluación, se realizará tras 5 minutos de suspender el aporte de oxígeno en caso de que el paciente esté recibiendo algún tipo de aporte. En caso de no ser posible suspender el aporte de O_2, la saturación se evaluará con el aporte actual de O_2 y se asignará una saturación menor a 90% para el punto final correspondiente.

Además, durante el seguimiento se evaluarán y registrarán en el eCRF: datos sobre terapias indicadas para manejo de COVID-19 (siempre que no sean tratamientos en el marco de una investigación), eventos clínicos (requerimiento de O2 >40%, necesidad de VNI o ventilación mecánica invasiva, pase a unidad de terapia intensiva – UTI, Infarto de miocardio, Accidente cerebrovascular y Tromboembolismo pulmonar) correspondientes a los puntos finales clínicos y los eventos adversos serios hasta los 30 días, el alta o la muerte, lo que ocurra primero. Toda la información de eventos será ingresada al

Confidencial

formulario de reporte de casos electrónicos (eCRF) que se diseñará para el estudio. La Figura 3 muestra el esquema de procedimientos del estudio.

Considerándose que se trata de un estudio independiente, pragmático, que no implica procedimientos asistenciales extraordinarios (para evitar sobrecargar a los equipos de salud en el marco de la pandemia, según las recomendaciones del Ministerio de Salud de la Nación), en el cual los datos a recolectar y registrar se obtendrán de la práctica asistencial de rutina en pacientes con COVID-19, todas las mediciones de signos vitales y saturación de O2 así como de calcemia, se llevarán a cabo con el equipamiento estándar que utiliza la institución, que son los aceptados y provistos por las autoridades jurisdiccionales para la práctica clínica habitual. Asimismo, la participación en el protocolo no interfiere ni impide la administración de cualquier terapia de sostén que se lleve a cabo por práctica habitual en los centros.

Todas las inconsistencias serán registradas y monitoreadas por el centro coordinador del estudio y se generarán consultas que se elevarán a cada investigador/a principal para su resolución.

Todos los eventos clínicos y eventos adversos serios, además de ser reportados al centro coordinador, deberán ser informados al Comité de Ética local de acuerdo a la regulación vigente.

Figura 3: Procedimientos del estudio.

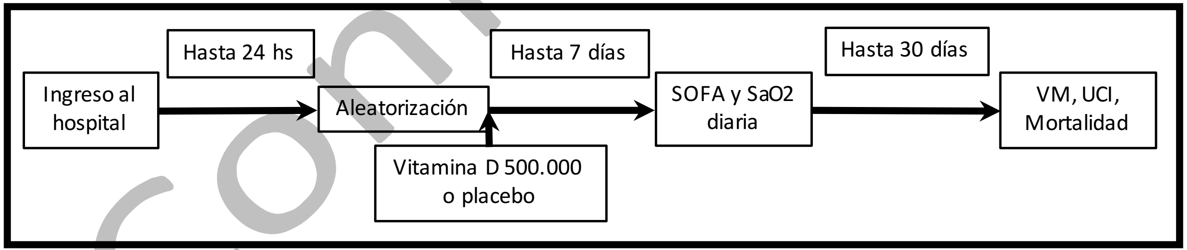

i. Doble ciego

La vitamina D se dispondrá en cápsulas blandas de 100.000 UI y el placebo será una cápsula idéntica en sus características organolépticas (forma, aspecto, color, sabor, olor y textura). Toda la medicación del estudio será envasada en el laboratorio de Farmacia de la Universidad Maimónides en frascos etiquetados con letra y el número de envase. Todos los frascos serán idénticos. Las listas de aleatorización serán almacenadas en el centro coordinador y la apertura del ciego solo será aceptada por solicitud del

Confidencial

investigador/a principal en casos de eventos serios para los cuales el conocimiento del tratamiento podría modificar la conducta clínica.

Los productos en investigación- principio activo y placebo- se elaborarán, manipularán y almacenarán según las Buenas Prácticas de Fabricación (BPF) de acuerdo con lo establecido por la Disposición 3287/18 de ANMAT. El principio activo será obtenido de una forma comercial (Deblax ®) y reenvasado para asegurar el ciego, mientras que el placebo será elaborado a los fines del protocolo en un laboratorio que cumpla con los requisitos de la mencionada normativa y reenvasado en la Farmacia Central de la Universidad.

j. Centros participantes

El Comité Ejecutivo del estudio identificará potenciales centros participantes. Tras la identificación, se establecerá contacto por vía de correo electrónico y/o teléfono con las/los referentes de los centros, para determinar el interés en participar. En caso de respuesta afirmativa se les enviará un formulario de factibilidad para determinar las posibilidades del centro de participar. (Ver Anexo CUESTIONARIO DE FACTIBILIDAD)

Se utilizarán como criterios la disposición a participar, la disponibilidad de recursos humanos para ser afectados al estudio y la cantidad de pacientes que potencialmente podría incluir cada centro.

En caso de ser seleccionado un centro, se procederá a la presentación del protocolo al equipo de investigación y luego su envío para la aprobación por el Comité de Ética en Investigación institucional. Tras la aprobación, se enviará la medicación del estudio y se completará el entrenamiento sobre los procedimientos del estudio en el centro. Las visitas de entrenamiento en los centros se realizarán de manera remota en los casos en los que no se pueda garantizar la distancia social.

Una vez que los centros cuenten con la aprobación de su CEI institucional, se conformará la lista de centros participantes en la Red multicéntrica CARED y sus Investigadores/as principales, la cual será informada a la totalidad de CEIs y autoridades institucionales.

k. Aleatorización

Se utilizará un esquema de aleatorización generada por computadora, estratificada, en bloques permutados y en una relación 1:1.

Confidencial

El esquema de aleatorización estratificada asegurará la distribución balanceada de variables pronósticas relevantes, y será por dos variables:

- Edad: igual o menor a 60 años o mayor a 60 años.
- Diabetes: si o no.

La medicación se preparará en bloques permutados de 8 tratamientos, que serán enviados a cada centro, de manera de asegurar una distribución balanceada de tratamientos en los estratos en cada uno de los sitios de estudio.

La asignación de los tratamientos será realizada por vía electrónica utilizando un sistema de respuesta interactiva basada en internet (IWRS, por sus siglas en inglés).

I. Cálculo del tamaño de la muestra

Para el primer objetivo de carácter cuantitativo por puntajes de la evolución del SOFA respiratorio se necesitarían 168 pacientes, 84 por grupo. Corregido por eventuales pérdidas por falta de compliance al protocolo, se estiman 200 pacientes en total.

Se planifica un análisis interino al alcanzar los 200 pacientes para evaluar si los resultados fueran positivos la ampliación de la muestra para objetivos clínicos.

En forma escalonada, de obtener un resultado positivo para el objetivo primario inicial, se incrementará la muestra para el objetivo de mayor repercusión clínica, que es evitar la oxigenoterapia y avance a asistencia respiratoria invasiva (Figura 4). Sobre la expectativa de una necesidad de oxigenoterapia de pacientes internados del 30% y un riesgo relativo de 0,75, es decir, una reducción del 25%, con un error alfa de 0,05 y un poder del 80% se requeriría un total de 1.072 pacientes, 536 por grupo. Ajustado por pérdidas la muestra necesaria se estima en 1.265 pacientes.

Figura 4. Etapas del protocolo

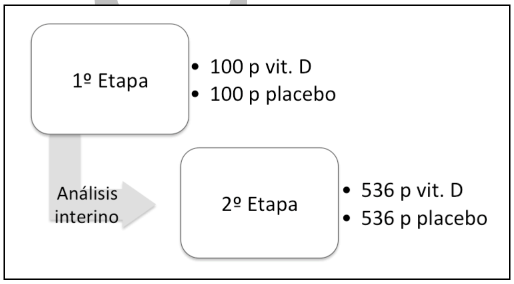

Confidencial

m. Reclutamiento de los pacientes

Se espera alcanzar el número de pacientes necesario para evaluar los resultados de la primera etapa a los 45 días de la activación del primer centro. Para esto se incluirán en esta etapa al menos 10 centros con casos de pacientes con COVID-19 en el período previo al inicio del estudio (Ver Anexo Listado de centros convocados a participar de la Red Multicéntrica del Ensayo CARED). De esta manera, cada centro debería incluir un promedio de 20 pacientes en este período de tiempo. En caso de una tasa de reclutamiento menor a la esperada se incluirán más centros para aumentarla. En total, se proyecta incluir al menos 15 centros en el estudio. Este número podría variar de acuerdo a los resultados de la primera etapa y a la tasa de reclutamiento en el estudio.

Para lograr cumplir el objetivo de obtener datos a los 60 días (y alcanzar 200 participantes a los 45 días) del inicio del estudio, se requiere incluir 4,4 pacientes por día (alrededor de 1 paciente cada 2 días en cada centro), lo cual estimamos como factible.

n. Metodología estadística

La descripción de datos cuantitativos será expresada como media y desvío standard cuando tengan distribución normal, o mediana e intervalos intercuartilos para datos no gaussianos o puntajes. Los datos cualitativos serán expresados como frecuencias absolutas y relativas. La comparación de las diferencias de los puntajes SOFA entre grupos será efectuada mediante el test U de Mann-Whitney. La incidencia de los puntos finales binarios será expresada como riesgos relativos con sus intervalos de confianza del 95% y las diferencias entre grupos serán evaluadas con el test de Chi^2 o el test Exacto de Fisher, según corresponda. Se derivarán del resultado los números necesarios de tratar y la modificación del riesgo absoluto. En la expresión final de los resultados se presentarán por subgrupos con test de interacción: grupos etarios, diabetes, hipertensión, sexo, epoc, antecedentes cardiovasculares, índice de masa corporal, lugar de residencia (villas de emergencia vs otras localizaciones), asma y tabaquismo. Para todos los cálculos se considerán como valor de p significativo <0,05. El análisis del punto final inicial y final serán efectuado con el criterio de intención de tratar.

o. Análisis interino

Se realizará un análisis interino tras la incorporación de los primeros 200 pacientes. Este análisis tendrá como objetivo evaluar los efectos del tratamiento sobre los puntos finales

Confidencial

de la primera etapa. La primera etapa está diseñada para tener un poder del 80% para detectar una diferencia de al menos un punto en el SOFA respiratorio. En caso detectarse una diferencia de esta magnitud o mayor, se procederá a continuar con la segunda etapa del estudio. En caso de diferencias de entre 1 y 0,3 puntos el Comité Ejecutivo del estudio discutirá con el Comité de Seguridad y Monitoreo de los Datos la estrategia a seguir. En esta discusión se tendrá en cuenta la magnitud de la diferencia, los efectos sobre otros puntos finales, el nivel de significancia y el poder eventual del estudio para detectar efectos de la intervención menor a los inicialmente planificados. El subanálisis fisiopatológico se realizará independientemente de los resultados de la primera etapa.

p. Seguridad del producto en estudio

Existe una vasta evidencia científica sobre el uso de una única dosis alta de vitamina D, carente de efectos tóxicos. Los reportes de intoxicaciones por exceso de vitamina D provienen del uso crónico en la mayor parte por megadosis.(30)

Un metaanálisis revisó sistemáticamente los 30 ensayos clínicos a 2014 que utilizaron dosis única elevada de vitamina D hasta 600.000 UI. Ninguno de los ensayos reportó efectos adversos fuera de intolerancia digestiva en pocos casos. Observaron hipercalciuia leve pero en ningún ensayo incremento de la incidencia de cálculos renales, hipercalcemia significativa ni daño renal. (31)

El ensayo controlado dirigido por el National Institute of Health estadounidense, posterior a este metaanálisis confirmó los mismos hallazgos, es decir, la ausencia de efectos adversos significativos. Evaluaron 1078 pacientes con sepsis, a los que se administró una dosis de vitamina D de 540.000 UI contra placebo. No observaron diferencias en la incidencia de hipercalcemia, insuficiencia renal ni cálculos renales (hubo sólo tres en el grupo placebo y ninguno en el grupo Vitamina D).(32)

En nuestro protocolo, con una carga única de 500.000 UI, y en base a la evidencia demostrada, si bien elevaríamos significativamente los niveles de vitamina D circulante -objetivo central- nos encontraríamos lejos de los niveles con evidencia de toxicidad, en forma similar a todos los ensayos que utilizaron dosis única elevada, como hemos documentado en la bibliografía resumida.

Durante el estudio se realizará un monitoreo y reporte de eventos adversos, de acuerdo con la regulación vigente y lo solicitado por el Comité de Ética institucional interviniente. El perfil de seguridad de la dosis y esquema (dosis única) del estudio es altamente seguro,

Confidencial

habiéndose reportado síntomas digestivos leves (dispepsia, náuseas). Además se reportaron hipercalcemias leves, hipercalciurias leves y aumento del magnesio urinario. Todos las alteraciones reportadas fueron manifestaciones de laboratorio, sin sintomatología clínica (26,29,33,34). (Ver Anexo: Manual de Información del producto Colecalciferol)

El procotolo contará también con un seguro que cubrirá eventuales demandas.

q. Aspectos éticos

Dado se trata de un ensayo clínico Fase IV que implica el uso de un principio activo aprobado por la Agencia Nacional de Medicamentos, Alimentos y Tecnologías (ANMAT) considerando que la dosis propuesta es superior al uso habitual y en el contexto de una emergencia sanitaria, corresponde a una investigación de riesgo mayor al mínimo de acuerdo con lo establecido por el Decreto 3385/09 de la Provincia de Buenos Aires y la Resolución 2033/19 del Ministerio de Salud de la Ciudad Autónoma de Buenos Aires (CABA).

Asimismo, la investigación corresponde a un estudio sin interés comercial, dirigida por un investigador perteneciente a una institución académica y un equipo responsable avalado por instituciones académicas, de acuerdo a lo establecido por el dictamen técnico 001/2016 de la Comisión Conjunta de Investigaciones en Salud (CCIS) de la Provincia de Buenos Aires (35). Siguiendo los lineamientos éticos establecidos por la OMS (36) y las recomendaciones del Comité Central de Ética (CCE) en Investigaciones de la CABA (37), se contempla la toma de muestras biológicas destinadas a investigaciones futuras, por lo que –exclusivamente en aquellos centros seleccionados para participar del subestudio fisiopatológico- se obtendrá un consentimiento amplio para el uso de muestras y datos en investigaciones futuras sobre COVID-19.

El estudio se realizará cumpliendo la legislación sobre investigación en sujetos humanos vigente en el ámbito nacional y provincial. Además, todos los procedimientos del estudio cumplirán con los principios de la Declaración de Helsinki y las pautas del Consejo Internacional de Ciencias Médicas (CIOMS).

Confidencial

El protocolo será presentado para evaluación y aprobación por cada uno de los Comités de Ética en Investigación de los centros invitados a participar, o comités subrogantes si correspondiera.

Además, el protocolo será presentado ante la CCIS de acuerdo a lo establecido por la Ley 11.044 de la Provincia de Buenos Aires. También será registrado en el Registro de Investigaciones en Salud (ReNIS) y en la Plataforma para el Registro Informatizado de Investigaciones en Salud de la Ciudad Autónona de Buenos Aires (PRIISA.BA), de acuerdo a lo requerido por la Resolución 1480/11 del Ministerio de Salud de la Nación y la Resolución 1679/19 del Ministerio de Salud de la Ciudad Autónoma de Buenos Aires, respectivamente. Asimismo, será registrado en cada una de las jurisdicciones donde se incorporen centros a la implementación del protocolo, si correspondiera, en cumplimiento con las normativas provinciales vigentes.

Antes de proceder al relevamiento de datos, toma de muestras sanguíneas y administración de la medicación del estudio, los/as investigadores/as del centro le brindarán a cada sujeto – siempre que cumpla criterios de inclusión y no presente criterios de exclusión- una explicación de los procedimientos y objetivos del estudio y lo/a invitarán a participar. Para ello le entregarán la hoja de información del estudio y se le solicitará la firma de un formulario de consentimiento informado en presencia de un testigo, perteneciente al equipo asistencial del centro pero que deberá ser externo al equipo de investigación. En aquellas instituciones donde por las normas de aislamiento de pacientes COVID-19 no fuera posible retirar de la habitación el consentimiento informado en formato papel, el/la investigador/a ingresará acompañado/a de cualquier integrante del equipo asistencial (pero externo al equipo de investigación) - que oficiará como testigo del proceso de toma de consentimiento informado- y llevará consigo un solo original del formulario de consentimiento. Si el/la paciente acepta participar, se le pedirá que firme el formulario de consentimiento original ingresado por el/la investigador/a, el cual quedará en poder del paciente hasta que se le permita su externación o finalice el aislamiento. Si por la disposición física del lugar de internación del centro, el ingreso de una persona para oficiar de testigo implica un riesgo para la misma, el/la testigo podrá participar del proceso de toma de consentimiento informado en forma virtual, sea por vía telefónica o video llamada. En los centros seleccionados para el subestudio fisiopatológico, en presencia del testigo se le solicitará que brinde su consentimiento para participar en el subestudio y

Confidencial

para que se le extraigan la muestra de sangre,. Si el centro utilizare un segundo original del consentimiento, este podrá ser firmado por el/la investigador/a y el/la testigo en la misma fecha pero en una instancia diferida, y se insertará una aclaración al respecto en la historia clínica. En todos los casos el equipo de investigación agotará las instancias para constatar y evidenciar la firma del paciente en el formulario originalmente firmado, lo cual deberá hacerse por medio de una fotografía digital que se remitirá al testigo interviniente y al Comité Ejecutivo del estudio y cuya impresión se guardará en el Archivo Central del Estudio.

En cada centro el/la IP presentará una declaración jurada referida al respeto de los derechos del paciente y el registro en la historia clínica del proceso de toma de consentimiento.

De acuerdo a lo establecido por las pautas éticas y operativas para la evaluación ética de investigaciones relacionadas con COVID-19 emitidas por la autoridad sanitaria nacional, es pertinente aclarar que el presente estudio no compromete la respuesta a la emergencia sanitaria establecida por la pandemia, ni interfiere indebidamente en la atención de las personas afectadas ni en la labor del personal de salud. Asimismo, esta investigación tiene valor social y es relevante por la potencial aplicación de sus resultados a la atención de personas afectadas por la enfermedad, y el conocimiento que se busca obtener no podría ser generado por otros medios alternativos ni fuera de la situación de emergencia sanitaria.

Las alternativas descriptas en relación a la obtención del consentimiento informado verbal tienen características de excepcionalidad para los centros y/o los casos en que no fuera posible obtenerlo por escrito en el mismo acto. Tanto la autorización de consentimiento verbal para casos expcepcionales como la autorización de firmar un solo documento de consentimiento informado (cuyas copias sean remitidas mediante fotografía digital) forman parte del plan de minimización del riesgo para extremar las medidas tendientes a evitar el contagio y la diseminación de la enfermedad en el marco de esta investigación y están sujetas a la aprobación del CEI institucional, en función de las condiciones asistenciales y de aislamiento de cada centro.

Asimismo, la obtención del consentimiento verbal - en los centros o los casos en que no fuera posible obtenerlo por escrito en el mismo acto- es pertinente en el marco de la emergencia sanitaria, considerando que en algunos casos exigir un consentimiento escrito

Confidencial

puede suponer riesgo de contagio, de acuerdo a lo contemplado por las pautas CIOMS (38), las recomendaciones del CCE de la CABA y al documento de Orientación ética sobre cuestiones planteadas por la pandemia del nuevo coronavirus de la Organización Panamericana de la Salud (39).

En aquellos centros en los que por razones de dinámica y condiciones asistenciales el CEI institucional autorizare de manera excepcional la obtención del consentimiento verbal, el/la IP del centro deberá presentar una declaración jurada al CEI comprometiéndose a respetar las consideraciones éticas y los derechos de los sujetos de investigación, en particular todas las consideraciones referidas al proceso de toma de consentimiento informado.

3. Definiciones

- **Hipertensión arterial:** se considerará hipertensión arterial a los casos con presión arterial mayor a 140/90 mmHg al ingreso, que el paciente refiera tener diagnóstico de hipertensión arterial o estar medicado con drogas antihipertensivas.
- **Diabetes:** se considerará diabéticos a los pacientes que estén medicados con drogas antidiabéticas o tengan antecedentes de diabetes y reciban solo tratamiento dietético.
- **EPOC:** se considerará como portador de EPOC (enfermedad pulmonar obstructiva crónica) a pacientes que reciban tratamiento para dicha enfermedad con broncodialatadores en forma crónica.
- **Asma:** se considerará como asmáticos a los pacientes que refieran este antecedente.
- **Enfermedad cardiovascular:** se considerarán como portadores de enfermedad cardiovascular a los pacientes que refieran antecedentes de infarto de miocardio, insuficiencia cardíaca, angioplastia coronaria percutánea, cirugía de revascularización y/o reemplazo valvular previo.
- **Enfermedad cerebrovascular:** se considerará como portador de enfermedad cerebrovascular a los pacientes que refieran antecedentes de accidente cerebrovascular previo (con o sin secuela neurológica en el momento del ingreso al estudio).

Confidencial

- Obesidad: se considerará criterio de obesidad a los/as pacientes cuyo Índice de Masa Corporal sea ≥30 (estimado según peso referido en kilogramos dividido la altura referida en metros al cuadrado)
- **SOFA respiratorio:** se medirá como una escala de 4 puntos (Figura 5) en la que un mayor puntaje indica más deterioro. Se estima dividiendo la presión parcial de O_2 en sangre arterial (PaO_2) por la Fracción inspirada de O_2. La PaO_2 se estimará a partir de la saturación de O_2 por oximetría de pulso (25). El puntaje será calculado con los datos de FiO2 y Saturación de O_2 que informen los investigadores. Todas las mediciones de Saturación de O_2 se realizarán tras 5 minutos de cualquier modificación en el aporte de O_2 para que se alcance el estado estable.

Figura 5: SOFA respiratorio.

	1	2	3	4
PaO_2/FiO_2 (mm Hg)	<400	<300	<220	<100

-Saturación de O_2: la saturación de O_2 se utilizará para calcular la diferencia entre la saturación de O_2 al ingreso y la menor saturación de O_2 registrada durante la internación, respirando aire ambiente. Este cálculo se realizará como la resta para cada paciente y se compararán las diferencias de medias entre los grupos. En los pacientes en los que, por su situación clínica, no sea posible suspender el aporte de oxígeno, se atribuirá una saturación de 89%. Todos las mediciones de saturación por oximetría de pulso y tras 5 minutos de suspender el aporte de O_2 para alcanzar un estado estable (40).

- **Quick SOFA:** el puntaje quick SOFA (qSOFA) se evaluará al ingreso y de acuedo a las mediciones diarias. Contiene tres variables: hipontensión arterial (tensión arterial sistólica menor o igual a 100 mHg), taquipnea (frecuencia respiratoria igual o mayor a 22 respiraciones por minuto) y alteración del estado mental (Glasgow menor a 15 – cualquier grado de alteración mental indica un

Confidencial

Glasgow menor a 15). El puntaje es de 0 a 3, más puntos indican mayor riesgo (41). Para el punto final se calcularán para cada paciente el qSOFA al ingreso y el peor registrado durante la internación y se compararán las medias entre grupos.

- **Requerimiento de asistencia con oxigenoterapia de alto flujo (>40% de FIO_2), indicación de ventilación no invasiva o invasiva:** se considerará como la ocurrencia de este punto final en caso de que se inicie tratamiento con O_2 para lograr una fracción inspirada mayor al 40% (más de 5 litros por minuto de O_2 a través de cánula nasal o máscara facial), o se inicie ventilación mecánica no invasiva con presión positiva continua en la vía aérea (CPAP) o con presión positiva de dos niveles en la vía aérea (BiPAP) mediante cualquier máscara adaptable, o se inicie ventilación mecánica invasiva mediante intubación orotraqueal (o nasotraqueal).
- **Derivación a Cuidados intensivos por necesidades clínicas:** se considerará como la ocurrencia de este punto final en los casos en los que, el empeoramiento de las condiciones clínicas del paciente, haga que éste requiera ser ingresado a una Unidad de Cuidados Intesivos (UCI), de acuerdo al criterio del médico tratante.
- **Ingeso a ventilación mecánica invasiva:** se considerará la ocurrencia de este punto final a partir del inicio de ventilación mecánica a través de intubación orotraqueal o nasotraqueal.
- **Accidente cerebrovascular:** pérdida focal de una función neurológica causada por un evento isquémico o hemorrágico con síntomas residuales mayores de 24 horas o que lleva a la muerte.
- **Infarto de miocardio:** desarrollo de dolor precordial, nuevas ondas Q y elevación enzimática compatible con la IV definición de infarto.
- **Insuficiencia renal aguda:** (o reagudización de insuficiencia renal crónica): incremento del 50% de los niveles iniciales de creatinina durante la evolución.
- **Tromboembolismo pulmonar:** cuadro clínico sugestivo (disnea, descompensación hemodinámica) confirmado por angiografía invasiva o tomografía multicorte.
- **Días de internación total:** será la diferencia de tiempo en días desde el ingreso al egreso del hospital.

Confidencial

- **Días de internación en UCI:** será la diferencia en días desde el ingreso a la UCI hasta el egreso de la misma. En los pacientes en los que no se haya requerido UCI, este será asignado como 0.
- **Mortalidad:** se evaluará la muerte por cualquier causa desde la aleatorización hasta el alta o los 30 días, lo que ocurra primero.

4. Sesgos potenciales y minimización de los riesgos

El diseño aleatorizado y doble ciego minimiza el sesgo por confusión. Se realizará una aleatorización estratificada para asegurar la distribución balanceada de dos variables de gran peso pronóstico como son la edad y la diabetes.

Los criterios de selección amplios están destinados a incluir la mayor cantidad de pacientes con un inicio de enfermedad no complicada aunque con riesgo de desarrollar complicaciones respiratorias. De esta manera se intenta reducir el riesgo de sesgo de selección que además podría afectar el poder del estudio.

Otra fuente de sesgos potencial es la pérdida en el seguimiento (atrición), para minimizarlo se simplificó el esquema terapéutico en estudio y se limitó el seguimiento al período intrahospitalario.

Otros sesgos como evaluación diferencial o reporte diferencial serán minimizados con el diseño doble ciego.

Para evaluar los puntos finales previstos en cada una de las etapas del ensayo, los pacientes serán aleatorizados y la evaluación de los eventos se realizará de manera ciega al tratamiento asignado, con el fin de reducir eventuales sesgos de selección o medición. Mediante la aleatorización y a través de la muestra estimada, se espera que la distribución entre ambos grupos se equilibre, tanto en lo relacionado con características de la población (sexo, antecedentes, etc) como en lo que se refiere a los eventuales tratamientos concomitantes. Para asegurar la distribución homogénea de la edad y la diabetes entre los grupos, se utiliza un esquema de aleatorización estratificado (ver la sección “Aleatorización”.

El ensayo no tiene entre sus objetivos evaluar el efecto de otros tratamientos o intervenciones, no obstante está previsto en el Formulario de Reporte de Casos (eCRF) el registro de todos los tratamientos que reciban los sujetos enrolados en el ensayo, por lo cual a criterio de los Comités de Seguridad de Datos y Comité Ejecutivo, podrán

Confidencial

realizarse los análisis de datos pertinentes para elucidar efectos posiblemente asociados a otros tratamientos.

5. Resultados esperados y divulgación de los resultados

El estudio evalúa un tratamiento de bajo costo, en una dosis única y con un excelente perfil de seguridad que tiene propiedades inmunomoduladoras en la respuesta antiviral. En caso de ser efectiva se podría disponer de un tratamiento ampliamente accesible a nivel lo cual tendría una gran relevancia sanitaria en el contexto de la pandemia por coronavirus.

Los resultados del estudio serán divulgados en la comunidad médica mediante su publicación en revistas biomédicas con arbitraje por pares y comunicaciones científicas.

Los resultados se publicarán de manera global, sin datos personales que pudieran identificar a los participantes según lo que se establece en la legislación vigentes respecto a la confidencialidad e intimidad de los sujetos participantes en investigaciones.

Confidencial

6. Bibliografía

[1]. Disponible en https://coronavirus.jhu.edu/data/mortality, accedido el 2/05/2020.

[2]. Hoffmann M, Kleine-Weber H, Schroeder S, Kruger N, Herrler T, Erichsen S, Schiergens T, Herrler G, Wu NH, Nitsche A, Muller MA, Drosten Ch, Pohlmann S. SARS-CoV-2 Cell Entry Depends on ACE2 and TMPRSS2 and Is Blocked by a Clinically Proven Protease Inhibitor. Cell 2020: 181, 1–10

[3]. Kuba, Y. Imai, S. Rao, H. Gao, F. Guo, B. Guan, Y. Huan, P. Yang, Y. Zhang, W. Deng, L. Bao, B. Zhang, G. Liu, Z. Wang, M. Chappell, Y. Liu, D. Zheng, A. Leibbrandt, T. Wada, A.S. Slutsky, D. Liu, C. Qin, C. Jiang, J.M. Penninger, A crucial role of angiotensin converting enzyme 2 (ACE2) in SARS coronavirus-induced lung injury, Nat. Med. 11 (2005) 875-879.

[4]. M. Vaduganathan, O. Vardeny, T. Michel, J. J.V. McMurray, M. A. Pfeffer, S. D. Solomon, Renin–Angiotensin–Aldosterone System Inhibitors in Patients with Covid-19, The New England Journal of Medicine. (2020). https://doi.org/10.1056/NEJMsr2005760.

[5]. M.L. Sun, J.M. Yang, Y.P. Sun, G.H. Su, Inhibitors of RAS Might Be a Good Choice for the Therapy of COVID-19 Pneumonia, Zhonghua Jie He He Hu Xi Za Zhi. 43(2020):E014. https://doi.org/10.3760/cma.j.issn.1001-0939.2020.0014.

[6] H. Cheng, Y. Wang, G.Q. Wang, Organ-protective Effect of Angiotensin-converting Enzyme 2 and its Effect on the Prognosis of COVID-19, J. Med. Virol. 2020. https://doi.org/10.1002/jmv.25785.

[7]. A.H.J. Danser, M. Epstein, D. Batlle, Renin-Angiotensin System Blockers and the COVID-19 Pandemic: At Present There Is No Evidence to Abandon Renin-Angiotensin System Blockers, Hypertension. 2020. https://doi.org/10.1161/HYPERTENSIONAHA.120.15082.

[8]. Kuba, Y. Imai, S. Rao, H. Gao, F. Guo, B. Guan, Y. Huan, P. Yang, Y. Zhang, W. Deng, L. Bao, B. Zhang, G. Liu, Z. Wang, M. Chappell, Y. Liu, D. Zheng, A. Leibbrandt, T. Wada, A.S. Slutsky, D. Liu, C. Qin, C. Jiang, J.M. Penninger, A crucial role of angiotensin converting enzyme 2 (ACE2) in SARS coronavirus-induced lung injury, Nat. Med. 11 (2005) 875-879.

[9]. M. Ferder, F. Inserra, W. Manucha, L. Ferder, The world pandemic of vitamin D deficiency could possibly be explained by cellular inflammatory response activity induced by the renin-angiotensin system, Am. J. Physiol. Cell. Physiol. 304 (2013) C1027-39.

[10]. D.A. Jolliffe, C.J. Griffiths, A.R. Martineau, Vitamin D in the prevention of acute respiratory infection: systematic review of clinical studies, J. Steroid. Biochem. Mol. Biol. 136 (2013) 321-329. https://doi.org/10.1016/j.jsbmb.2012.11.017.

[11]. J. Charan, J.P. Goyal, D. Saxena, P. Yadav, Vitamin D for prevention of respiratory tract infections: A systematic review and meta-analysis, J. Pharmacol. Pharmacother. 3 (2012) 300-303. https://doi.org/10.4103/0976-500X.103685.

[12]. A.R. Martineau, D.A Jolliffe, L. Greenberg, J.F. Aloia, P. Bergman, G. Dubnov-Raz, S. Esposito, D. Ganmaa, A.A. Ginde, E.C. Goodall, C.C. Grant, W. Janssens, M.E. Jensen, C.P.

Confidencial

Kerley, I. Laaksi, S. Manaseki-Holland, D. Mauger, D.R. Murdoch, R. Neale, J.R. Rees, S. Simpson, I. Stelmach, G. Trilok Kumar, M. Urashima, C.A. Camargo, C.J. Griffiths, R.L. Hooper, Vitamin D supplementation to prevent acute respiratory infections: individual participant data meta-analysis, Health Technol. Assess. 23 (2019) 1-44. https://doi.org/10.1136/bmj.i6583.

[13]. E.C. Goodall, A.C. Granados, K. Luinstra, E. Pullenayegum, B.L. Coleman, M. Loeb, M. Smieja, Vitamin D3 and gargling for the prevention of upper respiratory tract infections: a randomized controlled trial, BMC Infect. Dis. 19 (2014) 14:273. https://doi.org/10.1186/1471-2334-14-273.

[14]. D.A. Jolliffe, L. Greenberg, R.L. Hooper, C. Mathyssen, R. Rafiq, R.T. de Jongh, C.A. Camargo, C. J. Griffiths, W. Janssens, A.R. Martineau, Vitamin D to prevent exacerbations of COPD: systematic review and meta-analysis of individual participant data from randomised controlled trials, Thorax 74 (2019) 337–345. http://dx.doi.org/10.1136/thoraxjnl-2018-212092.

[15]. C. Cui, P. Xu, G. Li, Y. Qiao, W. Han, C. Geng, D. Liao, M. Yang, D. Chen, P. Jiang, Vitamin D receptor activation regulates microglia polarization and oxidative stress in spontaneously hypertensive rats and angiotensin II-exposed microglial cells: Role of renin-angiotensin system, Redox Biol. 26 (2019) 101295. https://doi.org/10.1016/j.redox.2019.101295.

[16]. A. Turin, J.J. Bax, D. Doukas, C. Joyce, J.J. Lopez, V. Mathew, G. Pontone, F. Shah, S. Singh, D.J. Wilber, M.G. Rabbat, Interactions Among Vitamin D, Atrial Fibrillation, and the Renin-Angiotensin-Aldosterone System, Am. J. Cardiol. 122 (2018) 780–784. https://doi.org/10.1016/j.amjcard.2018.05.013.

[17]. D. Carrara, R.M. Bruno, A. Bacca, S. Taddei, E. Duranti, L. Ghiadoni, G. Bernini, Cholecalciferol treatment downregulates renin-angiotensin system and improves endothelial function in essential hypertensive patients with hypovitaminosis D, J. Hypertens. 34 (2016) 2199–2205. https://doi.org/10.1097/HJH.0000000000001072.

[18]. P.S. Leung, The Modulatory Action of Vitamin D on the Renin-Angiotensin System and the Determination of Hepatic Insulin Resistance, Molecules. 24 (2019) pii: E2479. https://doi.org/10.3390/molecules24132479.

[19]. Y.C. Li, G. Qiao, M. Uskokovic, W. Xiang, W. Zheng, J. Kong, Vitamin D: a negative endocrine regulator of the renin-angiotensin system and blood pressure, J. Steroid. Biochem. Mol. Biol. 89-90 (2004) 387-392. https://doi.org/10.1016/j.jsbmb.2004.03.004.

[20]. D. Santoro, D. Caccamo, S. Lucisano, M. Buemi, K. Sebekova, D. Teta, L. De Nicola L, Interplay of vitamin D, erythropoiesis, and the renin-angiotensin system, Biomed. Res. Int. 2015 (2015) 145828. https://doi.org/10.1155/2015/145828.

[21]. J. Xu, J. Yang, J. Chen, Q. Luo, Q. Zhang, H. Zhang, Vitamin D alleviates lipopolysaccharide-induced acute lung injury via regulation of the renin-angiotensin system, Mol. Med. Rep. 16 (2017) 7432–7438. https://doi.org/10.3892/mmr.2017.7546.

[22]. J. Kong, X. Zhu, Y. Shi, T. Liu, Y. Chen, I. Bhan, Q. Zhao, R. Thadhani, Y.C. Li, VDR attenuates acute lung injury by blocking Ang-2-Tie-2 pathway and renin-angiotensin system, Mol. Endocrinol. 27 (2013) 2116-2125. https://doi.org/10.1210/me.2013-1146.

Confidencial

[23]. Y. Shi, T. Liu, L. Yao, Y. Xing, X. Zhao, J. Fu, X. Xue, Chronic vitamin D deficiency induces lung fibrosis through activation of the renin-angiotensin system, Sci. Rep. 7 (2017) 3312. https://doi.org/10.1038/s41598-017-03474-6.

[24]. Grant W, Lahore H, McDonnell S, Baggerly C et. al. Vitamin D Supplementation Could Prevent and Treat Influenza, Coronavirus, and Pneumonia Infections. doi: 10.20944/preprints202003.0235.v1

[25]. Laird E, Kenny RA. Vitamin D deficiency in Ireland –implications for COVID-19. Results from the Irish Longitudinal Study on Ageing (TILDA). https://www.doi.org/10.38018/TildaRe.2020-05

[26]. Kearns MD, Alvarez JA, Tangpricha V. Large, single-dose, oral vitamin D supplementation in adult populations: a systematic review. Endocr Pract. 2014 Apr;20(4):341-51. doi: 10.4158/EP13265.RA.

[27]. Gimenez M, Inserra F, Tajer C, Mariani J, Ferder L, Manucha W. Lungs as Target of COVID-19 Infection: Protective Common Molecular Mechanisms of Vitamin D and Melatonin as a New Potential Synergistic Treatment. Life Sciences, 2020. Aceptado para publicación.

[28]. Pandharipande PP, Shintani AK, Hagerman HE, St Jacques PJ, Rice TW, Sanders NW, Ware LB, Bernard GR, Ely EW. Derivation and validation of Spo2/Fio2 ratio to impute for Pao2/Fio2 ratio in the respiratory component of the Sequential Organ Failure Assessment score. Crit Care Med. 37 (2009) 1317-21. doi: 10.1097/CCM.0b013e31819cefa9.

[29]. Premaor MO, Scalco R, da Silva MJ, Froehlich PE, Furlanetto TW. The effect of a single dose versus a daily dose of cholecalciferol on the serum 25-hydroxycholecalciferol and parathyroid hormone levels in the elderly with secondary hyperparathyroidism living in a low-income housing unit. J Bone Miner Metab. 26 (2008) 603-8. doi: 10.1007/s00774-008-0858-0.

[30]. Marcinowska-Suchowierska E, Kupisz-Urbanska M, Łukaszkiewicz J, Płudowski,P, Jones G. Vitamin D Toxicity–A Clinical Perspective. Front. Endocrinol. 2018; 9:550.

[31]. Kearns MD, Alvarez JE, Tangpricha V. Large, single-dose, oral vitamin d supplementation in adult populations: a systematic review Endocr Pract. 2014 Apr; 20(4): 341–351.

[32]. The National Heart, Lung, and Blood Institute PETAL Clinical Trials Network . Early High-Dose Vitamin D3 for Critically Ill, Vitamin D–Deficient Patients . N Engl J Med 2019;381:2529-40.

[33]. Rossini M, Gatti D, Viapiana O, Fracassi E, Idolazzi L, Zanoni S, Adami S. Short-term effects on bone turnover markers of a single high dose of oral vitamin D_3. J Clin Endocrinol Metab. 97 (2012) E622-6. doi: 10.1210/jc.2011-2448.

[34]. von Restorff C, Bischoff-Ferrari HA, Theiler R. High-dose oral vitamin D3 supplementation in rheumatology patients with severe vitamin D3 deficiency. Bone. 45 (2009) 747-9. doi: 10.1016/j.bone.2009.06.012.

[35]. Documento técnico Comisión Conjunta de Investigaciones en Salud 001/16. Disponible en: http://www.ms.gba.gov.ar/ssps/investigacion/DocTecnicos/TipoInvestigaciones-Identificacionriesgos.pdf. Accedido el 4/05/2020.

[36]. Orientación ética sobre cuestiones planteadas por la pandemia del nuevo coronavirus (COVID-19). OMS 2020.

[37]. Recomendaciones del CCE referidas al Consentimiento Informado en investigaciones sobre COVID 19. Disponible en: https://www.buenosaires.gob.ar/sites/gcaba/files/recomendaciones_del_cce_referidas_al_consentimiento_informado_en_investigaciones_sobre_covid_19.pdf. Accedido el 4/05/2020.

[38]. Organización Panamericana de la Salud y Consejo de Organizaciones Internacionales de las Ciencias Médica. Pautas éticas internacionales para la investigación relacionada con la salud con seres humanos, Cuarta Edición. Ginebra: Consejo de Organizaciones Internacionales de las Ciencias Médicas (CIOMS); 2016.

[39]. Orientación ética sobre cuestiones clave planteadas por el brote. Washington, DC: OPS; 2016. Disponible en: http://iris.paho.org/xmlui/bitstream/handle/123456789/28485/OPSKBR16002_spa.pdf?sequence=10&ua=1. Accedido el 4/05/2020.

[40]. Gruber P, Kwiatkowski T, Silverman R, Flaster E, Auerbach C. Time to equilibration of oxygen saturation using pulse oximetry. Acad Emerg Med. 2 (1995) 810-5.

[41]. Seymour CW, Liu VX, Iwashyna TJ, Brunkhorst FM, Rea TD, Scherag A, Rubenfeld G, Kahn JM, Shankar-Hari M, Singer M, Deutschman CS, Escobar GJ, Angus DC. Assessment of Clinical Criteria for Sepsis: For the Third International Consensus Definitions for Sepsis and Septic Shock (Sepsis-3). JAMA. 315 (2016) 762-74. doi: 10.1001/jama.2016.0288.
